# Human neuromuscular junction three-dimensional organoid models and the insight in motor disorders

**DOI:** 10.1093/jmcb/mjab046

**Published:** 2021-07-16

**Authors:** Kejing Zhang, Lei Bai, Wentao Xu, Chengyong Shen

**Affiliations:** Department of Neurobiology, The First Affiliated Hospital, Institute of Translational Medicine, School of Medicine, Zhejiang University, Hangzhou 310020, China; Department of Neurobiology, The First Affiliated Hospital, Institute of Translational Medicine, School of Medicine, Zhejiang University, Hangzhou 310020, China; Department of Neurobiology, The First Affiliated Hospital, Institute of Translational Medicine, School of Medicine, Zhejiang University, Hangzhou 310020, China; Department of Neurobiology, The First Affiliated Hospital, Institute of Translational Medicine, School of Medicine, Zhejiang University, Hangzhou 310020, China

**Keywords:** neuromuscular junction, organoid, motor disorder

## Abstract

The neuromuscular junction (NMJ), a peripheral synaptic connection between motoneurons and skeletal muscle fibers, controls movement. Dysregulation of NMJs has been implicated in various motor disorders. Because of their large size and easy accessibility, NMJs have been extensively investigated in the neuroscience field and have greatly contributed to our understanding of the fundamental principles of synapses in the central nervous system. Researchers have tried multiple ways to develop models to recreate NMJs. Rapid progress in the research and development of tissue-like organoids has made it possible to produce human NMJ three-dimensional (3D) models *in vitro*, providing an additional powerful strategy to study NMJs. Here, we introduce the most recent advances of human embryonic stem cell- or induced pluripotent stem cell-derived organoids to model 3D NMJs.

## Introduction

‘*Every breath you take and every move you make…*’ is the opening lyric from a popular rock song of the 1980s. Human breathing and movement are delicately controlled by neuromuscular junctions (NMJs) (Sanes and Lichtman, 2001; Ribchester and Mehta, 2021). Improper formation or dysfunction of NMJs has been implicated in various motor disorders, including myasthenia gravis (MG), congenital myasthenia syndrome, and amyotrophic lateral sclerosis (Li et al., 2018; Wang et al., 2018).

**Figure 1:**
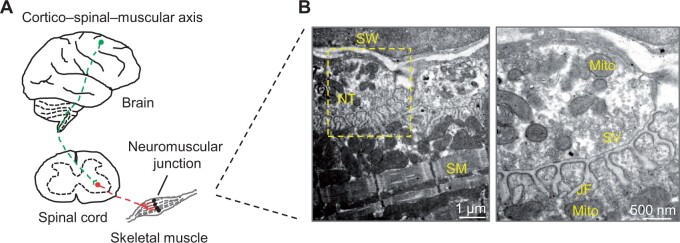
Motor circuits and NMJs. (**A**) Motor circuits consist of cortico–spinal–muscular axis. (**B**) A representative electron microscopy image of NMJs is shown. The boxed area is enlarged. JF, junctional fold; Mito, mitochondrion; NT, nerve terminal; SM, skeletal muscle; SV, synaptic vesicle; SW, Schwann cell.

In vertebrates, one mature NMJ is in the middle of each muscle fiber and occupies <0.1% of the muscle surface. In response to action potentials, motoneuron terminals release the neurotransmitter acetylcholine (ACh), which activates ACh receptors (AChRs) on the surface of muscle fibers to depolarize myotubes, thereby causing the release of calcium from the sarcoplasmic reticulum to trigger muscle contraction and movement (Wu et al., 2010). NMJs consist of three components: presynaptic motoneuron axon terminals, postsynaptic muscle fibers, and perisynaptic terminal Schwann cells (Darabid et al., 2014; Figure 1). On the presynaptic side of NMJs, nerve terminals are differentiated and exocytosis-competent synaptic vesicles are anchored at active zones. However, on the postsynaptic side, AChRs are extremely concentrated on the myotube membrane, compared with those in the non-synaptic region (density: 10000 vs. 10 per µm^2^). Presynaptic nerve terminals and the postsynaptic membrane align precisely to allow the efficient neuromuscular transmission. Between the presynaptic nerve terminal and the postsynaptic membrane, the synaptic cleft is filled with the synaptic basal lamina that anchors many critical proteins involved in NMJ development and maintenance.

Vertebrate NMJ development is regulated by agrin signaling. Motoneuron axon terminals release the ligand agrin to bind to low-density lipoprotein receptor-related protein 4 (LRP4) on myotube surfaces, which leads to the activation of muscle tyrosine kinase (MuSK), a transmembrane receptor. Activated MuSK transfers the extracellular signal into myotubes through the downstream adaptor Dok7, eventually inducing AChRs to cluster with the help of the scaffold protein Rapsyn (Tintignac et al., 2015; Li et al., 2018). Interestingly, although the agrin signaling is conserved between rodents and humans, the morphology of human NMJs looks different from that of rodent NMJs. Mouse NMJs exhibit unique ‘pretzel-like’ structures, while human NMJs are ‘nummular’ (appearing like coin-shaped patches). Compared with mouse NMJs, human NMJs are smaller, less complex, more fragmented, and more stabilized. Moreover, human NMJs have much thinner terminal axons and more rudimentary nerve terminals (Jones et al., 2017). These differences suggest that there might be unique characteristics of human NMJs.

Although animal models are powerful tools for NMJ study, they are expensive and time-consuming and sometimes cannot fully recapitulate human physiological and pathological phenotypes. Researchers have tried multiple ways to develop *in vitro* models to recreate NMJs as reviewed recently (Barbeau et al., 2020; Castellanos-Montiel et al., 2021). Owing to rapid progress in the research and development of pluripotent stem cell and tissue-like organoids, organoid-derived human NMJ three-dimensional (3D) models provide additional powerful tools to study NMJs. In the following sections, we will introduce the most recent advances of human embryonic stem cell (hESC)- or induced pluripotent stem cell (iPSC)-derived organoids to generate NMJ 3D *in vitro* models.

## Organoid-based human NMJ 3D models

**Figure 2:**
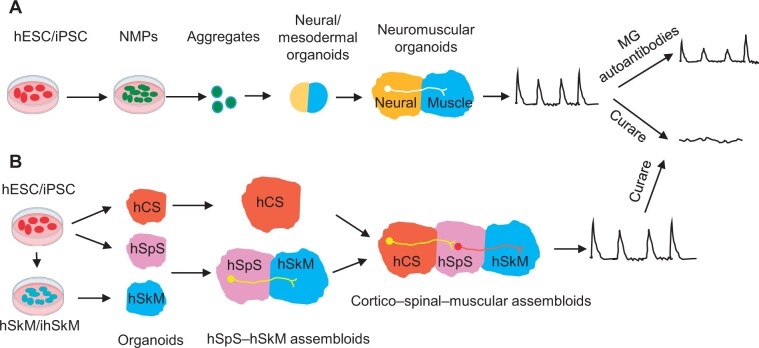
Human organoid-based NMJ 3D *in vitro* models. (**A**) Self-organization of neuromuscular organoids from NMPs is shown. (**B**) The assembly of cortico–spinal–muscular triple organoids is illustrated. Note that the neuromuscular organoid shown in **A** has a posterior spinal cord identity (posterior thoracic–lumbar identity), and the spinal cord organoid in the assembloid (shown in **B**) has an anterior identity (hindbrain to cervical spinal cord). hCS, human cortical spheroids; hSkM, human skeletal myoblasts; hSpS, human spinal spheroids; MG, myasthenia gravis; NMPs, neuromesodermal progenitors.

Organoids are self-organizing *in vitro* 3D tissue models containing multiple cell types similar to those in a specific tissue. In contrast to the monolayer in two-dimensional (2D) cell culture *in vitro*, organoids display greater cell diversity and remarkably organized tissue architecture. Thus, organoids are promising tools for tissue engineering and disease modeling (Brassard and Lutolf, 2019). As a result of recent rapid advances in pluripotent stem cell technology, organoids have been recently developed to mimic a number of human tissues, including the brain, intestines, kidneys, liver, and pancreas (Spence et al., 2011; Lancaster et al., 2013; Morizane et al., 2015; Pasca et al., 2015; Broutier et al., 2016). To date, generating human organoids composed of multiple tissues to reconstruct neurological circuits such as neuromuscular connections is still a challenge (Marton and Pasca, 2020). In 2019, the Lancaster laboratory adapted air–liquid interface culture of cerebral organoids. When cocultured with mouse spinal cord–muscle explants, human cerebral organoids functionally innervated the spinal cord and consequently elicited muscle contractions (Giandomenico et al., 2019). However, species differences between mice and humans might cause the failure in clinical trials of drug candidates, highlighting the importance of using cells that are all derived from human origin. Recently, the Gouti group and the Pasca group used different elegant strategies to successfully generate human NMJ 3D organoid models *in vitro* (Figure 2; Andersen et al., 2020; Faustino Martins et al., 2020). Faustino Martins et al. (2020) from the Gouti laboratory used a single progenitor population to generate a complex organoid of the spinal cord and skeletal muscle, leading to self-organization of neuromuscular organoids, while Andersen et al. (2020) from the Pasca laboratory fused three different tissue organoids to generate cortico–spinal–muscular assembloids.

### Neuromuscular organoids

Accumulated evidence has demonstrated that the posterior spinal cord and associated muscles originate from a common stem cell population called neuromesodermal progenitors (NMPs) (Henrique et al., 2015). Because they are committed to forming both mesodermal and neuroectodermal cells but not endodermal derivatives, NMPs would be the appropriate starting cells to generate neuromuscular organoids. Based on this idea, Faustino Martins et al. (2020) recently used hESC/iPSC-derived NMPs to self-organize the neuromuscular 3D organoids *in vitro*. First, hESCs or human iPSCs were treated with Wnt/FGF factors to generate an efficient cell number of NMPs in the 2D culture. Next, the NMPs were transferred to round-bottomed plates with ultralow adhesion to form 3D cell aggregates. Then the NMPs further proliferated and differentiated simultaneously into cells that had restricted neural and mesodermal fates, which separated spatially in the neuromuscular organoids. The two lineages interacted during their maturation and self-organized to form functional NMJs comprising spinal cord neurons, skeletal myotubes, and terminal Schwann cells. The expression of HOX genes revealed that the neuromuscular organoids had maintained their initial HOX code corresponding to a posterior spinal cord identity. This is particularly important because most of lower motor neurons are from the posterior spinal cord. Single-cell analysis further revealed that both neural and mesodermal lineages were generated in their NMP 3D organoids. Notably, the cellular composition and developmental features were reproducible in each organoid, suggesting the high consistency among different batches of experiments and different cell lines.

In the neural part of Day 50 neuromuscular organoids, electron microscopy analysis revealed the presence of densely packed longitudinal axons, synaptic clefts, and synaptic vesicles in neurons. Myelinated axons expressing myelin basic protein were also detected. In the neural part close to the skeletal muscle, choline acetyltransferase (ChAT), an ACh-synthesizing enzyme, was clustered. There were parallel-aligned actin filaments, distinct Z-lines, and M-bands in muscle regions, suggesting that mature sarcomeres had formed in the neuromuscular organoids. Strikingly, numerous AChR clusters along the contact sites between neurites and myotubes were labelled by α-bungarotoxin (α-BTX), a peptide that specifically binds to AChRs with a high affinity, indicating the formation of NMJs. Electron microscopy revealed the presence of synaptic vesicles in presynaptic nerve terminals and junctional fold-like structures on the muscle basement membrane. S100β-positive terminal Schwann cells capped the neuronal terminals. Thus, all three cellular components required for the development of functional NMJs were generated and self-organized in the neuromuscular 3D organoids. The number of AChR clusters per muscle fiber declined from Day 50 to Day 100 but was maintained after Day 150, similar to the dynamic remodeling of NMJs during development but stabilizing at the adult stage *in vivo*. Notably, neuromuscular organoids developed the nerve-controlled contractile activity of muscles, which was effectively blocked by curare, a neurotoxin specifically and competitively blocking AChRs, demonstrating that muscle activity was dependent on functional NMJs in the neuromuscular organoids. Note that neuromuscular organoids produced a complex spinal neural network similar to central pattern generator circuits, which produce rhythmic signals critical for walking and breathing.

### Cortico–spinal–muscular triple organoids

In contrast to the self-organization of spinal cord and muscle-like tissues from a single progenitor population to generate neuromuscular organoids, the Pasca group recently used a different method to assemble cortico–spinal–muscular triple organoids in one dish (Andersen et al., 2020).

Using human iPSCs, Andersen et al. (2020) first generated human spinal spheroids (hSpS) resembling the hindbrain/cervical spinal cord with an anterior identity. hSpS expressed the motoneuron progenitor marker Oligo2 and the motoneuron marker ChAT sequentially. To investigate whether hSpS could mediate muscle contraction, limb buds were dissected from embryonic day 11.5 mouse embryos before spinal motoneuron innervation, and the limb buds were then directly cocultured with hSpS. The limb buds exhibited spontaneous contractions when assembled with hSpS but not when cultured alone. The activity of contractions in hSpS–limb assembloids persisted for at least 2 weeks *in vitro* and was completely blocked by the addition of curare. The ability of hSpS to regulate the activity of human skeletal myoblasts (hSkM) derived from adult muscle biopsies was also tested. 3D hSkM were generated by adding dissociated proliferative hSkM to an extracellular matrix in a silicone well to differentiate into mature multinucleated myotubes, as evidenced by the expression of skeletal muscle markers including Desmin, Titin, and MyHC. To assemble hSpS–hSkM organoids, an hSpS organoid was placed closely near a 3D hSkM organoid on top of a 6-well transwell insert. Approximately 2 weeks later, abundant neurons from hSpS projected to the 3D hSkM. Live imaging with the calcium indicator Cal-590 revealed that the proportion of active hSkM doubled in hSpS–hSkM organoids in comparison to hSkM organoids alone, supporting the idea that motoneuron innervation promotes muscle maturation *in vivo*. Indeed, retrograde rabies virus tracing verified that most, if not all, of the projections from hSpS to hSkM were from ChAT-positive motoneurons but not GABA-positive interneurons in the hSpS. Using glutamate uncaging, they further showed that these hSpS cholinergic projections were functionally connected to hSkM. Muscle contractions in hSkM were blocked with curare treatment. Immunocytochemistry analysis also showed that the presence of NMJs by labelling α-BTX (the postsynaptic marker of NMJs) and synaptophysin (the presynaptic marker of NMJs) on Desmin-positive myotubes.

To reconstruct a complete motor circuit consisting of the cortico–spinal–muscular axis, Andersen et al. (2020) further assembled hSpS–hSkM 3D organoids with human cortical spheroids (hCS) on the top of transwell inserts. The hSC were generated as previously described (Pasca et al., 2015; Birey et al., 2017; Sloan et al., 2018). During Day 60–Day 120, hCS were assembled with hSpS. Transcriptional analyses and mapping suggested that after 2.5 months, hCS resembled the mid-fetal prenatal brain (19–24 weeks post-conception in humans). The hCS contain glutamatergic neurons from deep and superficial cortical layers alike. Superficial-layer neurons were confirmed by the expression of the homeodomain family proteins CUX1 and CUX2, mostly localized to layers II–IV. Cortico–spinal-associated markers FEZF2, BCL11B, and SOX5 were also expressed in hCS. These neurons were electrophysiologically mature, displayed spontaneous activity, were surrounded by nonreactive astrocytes, and formed functional synapses (Pasca et al., 2015; Andersen et al., 2020; Marton and Pasca, 2020). Impressively, Andersen et al. (2020) found that hCS–hSpS–hSkM triple assembloids could be kept in culture for up to 10 weeks after post-assembly without structural disintegration, exhibiting hCS-derived projections into hSpS, motoneurons, non-reactive astrocytes, myelinating oligodendrocytes, organized skeletal muscle fibers, and NMJs at morphological levels. Cortical activation-induced hSkM contractions in hCS–hSpS–hSkM assembloids, which were functionally verified by optogenetic stimulation. Excitingly, hCS–hSpS–hSkM assembloids can be prepared entirely from human iPSCs, which has increased the opportunity for modeling motor disorders. Although the success rate in response to optogenetic stimulation for contracting assembloids was reduced after 2 months of culture, light-induced activity of hCS-triggered calcium spikes in hSkM maintained a robust response after consecutive stimulations, suggesting that cortico–spinal–muscular tripleassembloids can be maintained functionally *in vitro* over several weeks. This is important because myotubes are easily detached from the dish surface in the current NMJ *in vitro* models after long culture periods (Barbeau et al., 2020).

### Disease modeling

Human organoid-derived NMJ models contain functional NMJs and other key features. They are reproducible and can be maintained in culture for weeks to several months. Thus, organoid-derived NMJ models provide attractive *in vitro* NMJ 3D models to study neuromuscular disorders and develop potential therapies. MG is the most common neuromuscular disorder caused by autoantibodies aberrantly targeting the key synaptic molecules at NMJs such as AChRs, MuSK, or LRP4 (Dalakas, 2019; Gilhus et al., 2019). Faustino Martins et al. (2020) treated neuromuscular organoids with autoantibodies from MG patient serum to model the MG autoimmune disorder. Compared with control patient serum, treatment with MG patient serum reduced the number of AChR clusters and the muscle contraction rate and amplitude, recapitulating key features of the MG disease phenotype (Faustino Martins et al., 2020; Ichida and Ko, 2020). In addition to AChR, MuSK, LRP4, and agrin antibodies, >10 antibodies have been identified in MG patients, such as antibodies against Titin and Cortactin (Gilhus et al., 2016; Dalakas, 2019). These autoantibodies could be causative or a mere secondary effect due to the abnormal immunity or inflammation. Compared with traditional animal models of experimental autoimmune MG (EAMG), human neuromuscular organoids provide a convenient tool to examine the roles of human autoantibodies in the pathogenesis of MG, which is ultimately beneficial for the precise and quick diagnosis of seronegative MG patients. The key minimal domain in autoantigens that are specifically involved in the pathogenesis of MG could be identified using this model. Furthermore, although EAMG provides excellent animal models to study the pathological mechanisms of MG and develop treatments, it cannot replicate all the pathologies that occur in human patients (Wang et al., 2018). For example, human NMJs exhibit some morphological differences from rodent NMJs (Jones et al., 2017). Thus, human organoid-derived NMJ 3D models might have some advantages over rodent-based EAMG. Finally, other autoimmune diseases, such as Lambert–Eaton myasthenic syndrome, which is caused by autoantibodies attacking voltage-gated calcium channels at the presynapse (Gilhus et al., 2019), would also be worthy of testing in organoid-derived NMJ models.

## The advantages of human neuromuscular 3D organoids

Significant progress has been made in the generation of either spinal motoneurons or skeletal muscle fibers in 2D or 3D systems (Barbeau et al., 2020). Compared with traditional 2D coculture models, biomaterial-based NMJ 3D coculture models, organotypic slice culture models, and animal animals, organoid-derived human NMJ models have several advantages.

The conventional coculture system of motoneuron and muscle is not amenable to long-term experiments. Differentiated myotubes tend to detach from the dish surface, which causes functional mature muscle fibers to decrease over time. Human organoid-derived NMJ models can be maintained in culture for weeks to months (even surviving for 1 year) (Andersen et al., 2020; Faustino Martins et al., 2020), which provides a large time window to study NMJ development, maintenance, degeneration, and regeneration.

Compared with the monolayer culture of NMJ 2D models (Steinbeck et al., 2016) or biomaterial-based NMJ 3D models (Afshar Bakooshli et al., 2019; Ko et al., 2019; Osaki et al., 2020), organoid-derived NMJ models generate diverse cell types and form the interactions between cells in three dimensions, which is more similar to the native tissue architecture *in vivo.* The same population of cells (NMPs) gives rise to neural and muscle populations simultaneously and self-organizes into neuromuscular organoids. Single-cell analysis showed that the organoids reproducibly exhibited posterior identity. In cortico–spinal–muscular assembloids, neurotransmitter identity in the neuronal clusters included glutamatergic, GABAergic, glycinergic, and cholinergic cells.

NMJ development requires intimate interactions among nerve terminals, muscle fibers, and terminal Schwann cells. Their development is influenced commutatively (Darabid et al., 2014). Glial cells are usually absent in the previous 2D culture dish. Compared to the coculture of motoneurons and muscle fibers, neuromuscular organoids provide a timely interaction between motoneurons, muscle fibers, and even Schwann cells. Furthermore, NMJs are supported by terminal Schwann cells, which promote the formation and maturation of intrinsically functional NMJ networks. The formation of neuromuscular connections can be investigated at any time in the 3D microenvironment, as presented in embryos.

Cortico–spinal–muscular assembloids generate input from the motor cortex to the skeletal muscles. Neuromuscular organoids produce a spinal neural network that is similar to central pattern generator circuits. These results provide a broader application to study the motor circuits in the future.

Organotypic slice cultures *ex* vivo closely resemble that observed *in situ* (Gahwiler et al., 1997). They can be cultured for weeks to months, and thus are a very useful tool for experiments that require long-term survival of the preparation. Slice cultures are usually derived from early postnatal animals. However, it is very difficult to get young human samples for organotypic slice culture and also it requires strict ethic agreement to use fresh human samples. Using hESCs or iPSCs, organoid-derived system overcomes this shortcoming and shows potentials for studying human tissue development and disease modeling.

One of the most practical advantages of human organoid-derived NMJ models is disease modeling. At present, most human-related neuromuscular disorders are studied in animal models that may contribute to the low translational success rate of nervous system therapies. The development of *in vitro* human-specific models is therefore critical to overcome interspecies differences, capture human-specific neurobiological traits, and accelerate the process of drug screening. Thus, human organoid-derived NMJ models provide a useful approach that complements the use of animal models for studying disease.

## Perspectives

Organoid-based NMJ 3D models have exhibited promising potentials for studying mechanisms and disease modeling; however, some challenges remain in the field.

### Similarity in morphology

Remarkable changes occur during NMJ development. In rodents, after birth, neonatal plaque-like AChR clusters transform into multi-perforated elaborate branches and mature NMJs display a pretzel-like morphology at the adult stage (Shi et al., 2012). In human NMJs, mature NMJs exhibit nummular-like structures (Jones et al., 2017). Presynaptic axon terminals and postsynaptic AChR assembly are aligned tightly, which ensures the efficient neuromuscular transmission. In addition, excessive NMJs are eliminated during early development; however, one mature NMJ is maintained in the middle of each myotube for proper motor controls. Muscle fiber membrane at the postsynaptic region forms deep invaginations called junctional folds, which are believed to expand the synapse area (Li et al., 2018). Similar to previous *in vitro* NMJ models, organoid-based NMJ models exhibit scattered α-BTX-positive AChR clusters along each myotube. The junctional folds in NMJ organoids exhibit protrusion-like structure. A better understanding of the mechanisms underlying AChR prepattening in the muscle before innervation would help explain how NMJs form only at the synaptic region. The presence of extracellular matrix at the postsynaptic membrane is also critical for the development and maintenance of the NMJ structure.

### Spatiotemporal control

Human spinal motoneuron somas are restricted in the ventral hood of the spinal cord, while their axons extend a long distance to innervate skeletal muscle fibers. Microfluidics used in previous NMJ models allow spinal motoneurons and muscle cells to grow in different chambers that are connected by axons (Altman et al., 2019). This provides convenience to study axon (such as local translation and axonal transport) and somas separately without interfering from each other. The combination of microfluidic chambers with organoid culture would make it possible to locally study soma, axons, or NMJs.

### Disease modeling

In addition to NMJ autoimmune disorders, other motor disorders caused by genetic mutations, such as congenital myasthenia syndrome, amyotrophic lateral sclerosis, Charcot–Marie–Tooth disease, spinal muscular atrophy, and muscular dystrophy, could also be investigated using organoid-derived NMJ models. A complete patient-derived organoids or a combination of healthy and patient-derived organoids could provide new insights into disease mechanisms such as dissecting cell-specific vulnerabilities and cell-autonomous effects of these disorders. Human organoid-derived NMJ models also provide an ideal platform to screen novel drugs for aforementioned diseases on a large scale in the future.

In general, human organoid system showed great potentials for tissue engineering and disease modeling. However, as an *in vitro* tool, we have to realize that there is some distance from real human tissues *in vivo*. Using proper controls to ensure functional organoids in each batch of experiments would be important. Also, it relies on further technique development of organoid culture to better mimic tissues *in vivo.* Organoid-derived human NMJ 3D models could be a complementary tool of 2D cell culture system, animal models, as well as human samples, to study synapses and motor disorders.
